# The most effective but largely ignored target for prostate cancer early detection and intervention

**DOI:** 10.7150/jca.72973

**Published:** 2022-10-17

**Authors:** Yan-gao Man, Ciaran Mannion, Anahid Jewett, Yi-Hsuan Hsiao, Aijun Liu, Andrzej Semczuk, Paul Zarogoulidis, Andrei B. Gapeev, Alessia Cimadamore, Peng Lee, Antonio Lopez-Beltran, Rodolfo Montironi, Francesco Massari, Xin Lu, Liang Cheng

**Affiliations:** 1 Department of Pathology, Hackensack Meridian School of Medicine, Nutley, NJ, USA; 2 Tumor Immunology Laboratory, Jonsson Comprehensive Cancer Center, UCLA School of Dentistry and Medicine, Los Angeles, CA, USA; 3 Department of Obstetrics and Gynecology, Changhua Christian Hospital, Changhua, Taiwan; 4 Department of Pathology, Chinese PLA General Hospital 7 th Medical Center, Beijing, China; 5 II ND Department of Gynecology, Lublin Medical University, Lublin, Poland; 6 Pulmonary-Oncology Department, "Theageneio" Cancer Hospital, Thessaloniki, Greece; 7 Laboratory of Biological Effects of Non-Ionizing Radiation, Institute of Cell Biophysics, Russian Academy of Sciences, Russian Federation; 8 Section of Pathological Anatomy, Polytechnic University of the Marche Region, School of Medicine, United Hospitals, Ancona, Italy.; 9 Department of Pathology, New York University School of Medicine, New York, NY, USA.; 10 Department of Pathology, New York Harbor Healthcare System, New York, NY, USA.; 11 Department of Morphological Sciences, Cordoba University Medical School, Cordoba, Spain; 12 Molecular Medicine and Cell Therapy Foundation, Department of Clinical & Molecular Sciences, Polytechnic University of the Marche Region, Ancona, Italy; 13 Medical Oncology, IRCCS Azienda Ospedaliero-Universitaria di Bologna, Bologna, Italy; 14 Department of Biological Sciences, Boler-Parseghian Center for Rare and Neglected Diseases, Harper Cancer Research Institute, University of Notre Dame, Notre Dame, IN, USA.; 15 Tumor Microenvironment and Metastasis Program, Indiana University Melvin and Bren Simon Comprehensive Cancer Center, Indianapolis, IN, USA.; 16 Department of Pathology and Laboratory Medicine, Brown University Medical School | Lifespan Academic Medical Center, RI, USA.

**Keywords:** Prostate basal cell layer, Tumor capsule, Cancer early detection and intervention.

## Abstract

Over the past two decades, the global efforts for the early detection and intervention of prostate cancer seem to have made significant progresses in the basic researches, but the clinic outcomes have been disappointing: (1) prostate cancer is still the most common non-cutaneous cancer in Europe in men, (2) the age-standardized prostate cancer rate has increased in nearly all Asian and African countries, (3) the proportion of advanced cancers at the diagnosis has increased to 8.2% from 3.9% in the USA, (4) the worldwide use of PSA testing and digital rectal examination have failed to reduce the prostate cancer mortality, and (5) there is still no effective preventive method to significantly reduce the development, invasion, and metastasis of prostate cancer… Together, these facts strongly suggest that the global efforts during the past appear to be not in a correlated target with markedly inconsistent basic research and clinic outcomes. The most likely cause for the inconsistence appears due to the fact that basic scientific studies are traditionally conducted on the cell lines and animal models, where it is impossible to completely reflect or replicate the *in vivo* status. Thus, we would like to propose the human prostate basal cell layer (PBCL) as “the most effective target for the early detection and intervention of prostate cancer”. Our proposal is based on the morphologic, immunohistochemical and molecular evidence from our recent studies of normal and cancerous human prostate tissues with detailed clinic follow-up data. We believe that the human tissue-derived basic research data may provide a more realistic roadmap to guide the clinic practice and to avoid the potential misleading from *in vitro* and animal studies.

## Editorial Commentary

Over the past two decades, a wide variety of genetic and epigenetic factors, including the germline variants, somatic gene mutations, various SNPs and miRNAs, aberrant expression of tumor suppressor genes or oncogenes, tumor regulatory drivers or nominate novel transcription factors, phosphorylation, glycosylation, ubiquitination, acetylation, lipidation, bacterial or viral infections, dietary compounds, or changes in testosterone-estradiol ratio…, have been added to the list of risk factors for prostate carcinogenesis and progression 1-21. Concurrently, a wide variety of detection and intervention methods, including artificial intelligence or nanotechnology -based devices, MRI-guided biopsy, bispecific antibodies, cancer vaccines, cytokine inhibitions, chimeric antigen receptor T-cells, immune checkpoint inhibition, high-intensity focal ultrasound, focal cryotherapy, photodynamic therapy, different sources of energy-based focal therapy, focal laser ablation, irreversible electroporation, DNA PSA-specific aptamers…, have been developed for clinic applications 22-32.

Unfortunately, the outcomes of global efforts for the early detection and intervention of prostate cancer are disappointing: (1) prostate cancer is still the most common cancer in Europe in man, (2) the prostate cancer incidence has increased in nearly all African and Asian countries, (3) the proportion of advanced cancers at diagnosis has increased from 3.9% to 8.2% in the US, (4) the worldwide use of PSA testing and digital rectal examination has failed to reduce prostate cancer mortality, and thus, is no longer recommended 33-44, and (5) there is still no effective preventive method to reduce the incidence, invasion, and metastasis of prostate cancer 22-32.

Together, these facts suggest that global efforts during the past appear to be not in a correlated target. The most likely cause appears due to the fact that basic scientific studies are traditionally conducted on the cell lines and animal models, which are impossible to completely reflect or replicate the *in vivo* status. Thus, we propose the prostate basal cell layer (PBCL) as “the most effective target for the early detection and intervention of prostate cancer” for the following reasons.

## The PBCL is an essential component of the prostate gland

**Figure 1:**
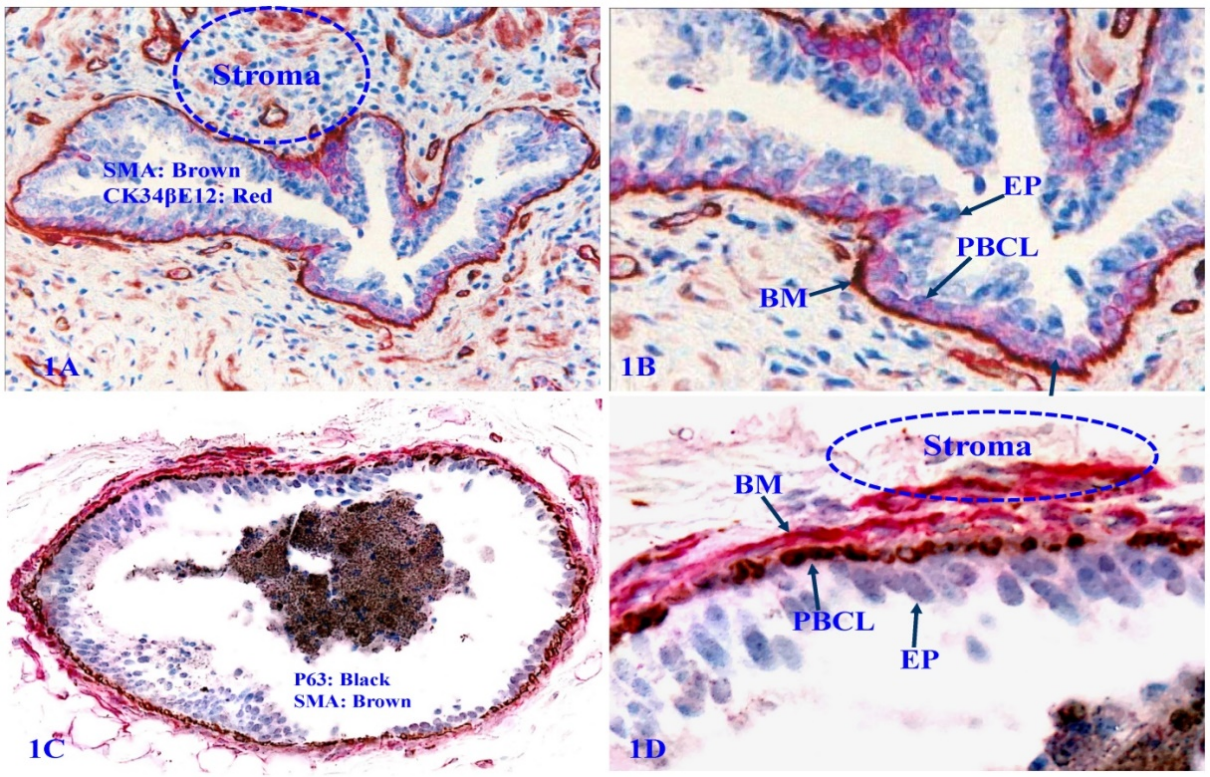
** Structural relationships of prostate gland tissue components.** Formalin-fixed and paraffin-embedded human prostate gland tissue sections were double immune-stained, 1A and 1B with antibodies to smooth muscle actin (SMA) that is reactive to the basement membrane (BM), smooth muscle, and endothelial cells, and CK34βE12, which is reactive to PBCL; 1C and 1D with SMA plus p63, which is reactive to PBCL. EP = Epithelium.

The human prostate gland consists of three components: the epithelium, capsule, and stroma. The epithelium is completely encircled by the capsule (a single layer of PBCL) and the basement membrane (BM, a thin sheet of extracellular fibers), and the stroma. Therefore, prostate cancer cells have to first pass through the PBCL and then BM to invade to the stroma 45-49 (Figure 1).

## The PBCL is the source of several tumor suppressors

**Figure 2:**
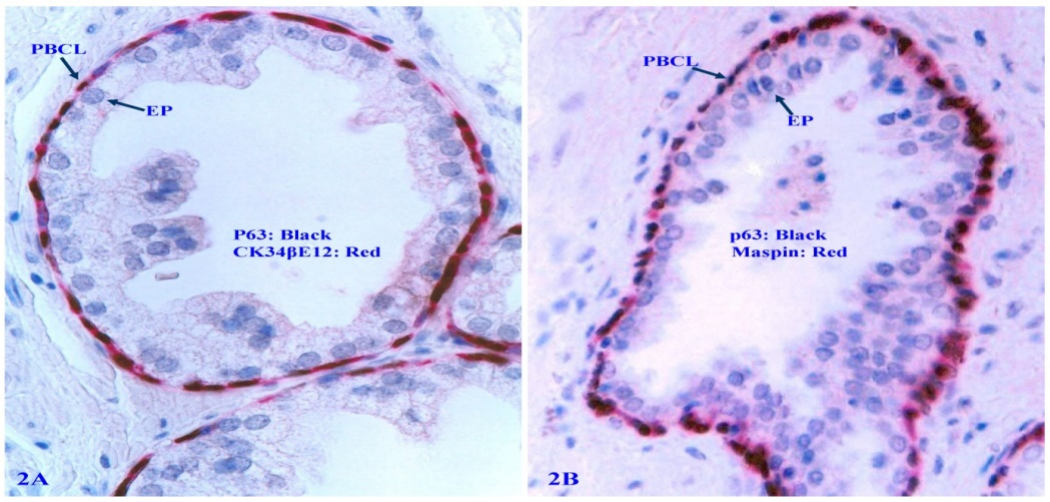
** Expression of tumor suppressors p63 and Maspin in PBCL.** Formalin-fixed and paraffin-embedded human prostate gland sections were double immune-stained, 2A with CK34βE12 (red) and p63 (black); 2B with Maspin (red) and p63 (black). Please note that the normal PBCL is continuous and the basal cells express a high level of Maspin and p63. PBCL = Prostate basal cell layer. EP = Epithelium.

The PBCL produces several tumor suppressors, including Maspin and p63 50-54 that exert significant regulatory functions on adjacent epithelial cells (Figure 2).

## The focal PBCL degeneration is a triggering factor for tumor progression

**Figure 3:**
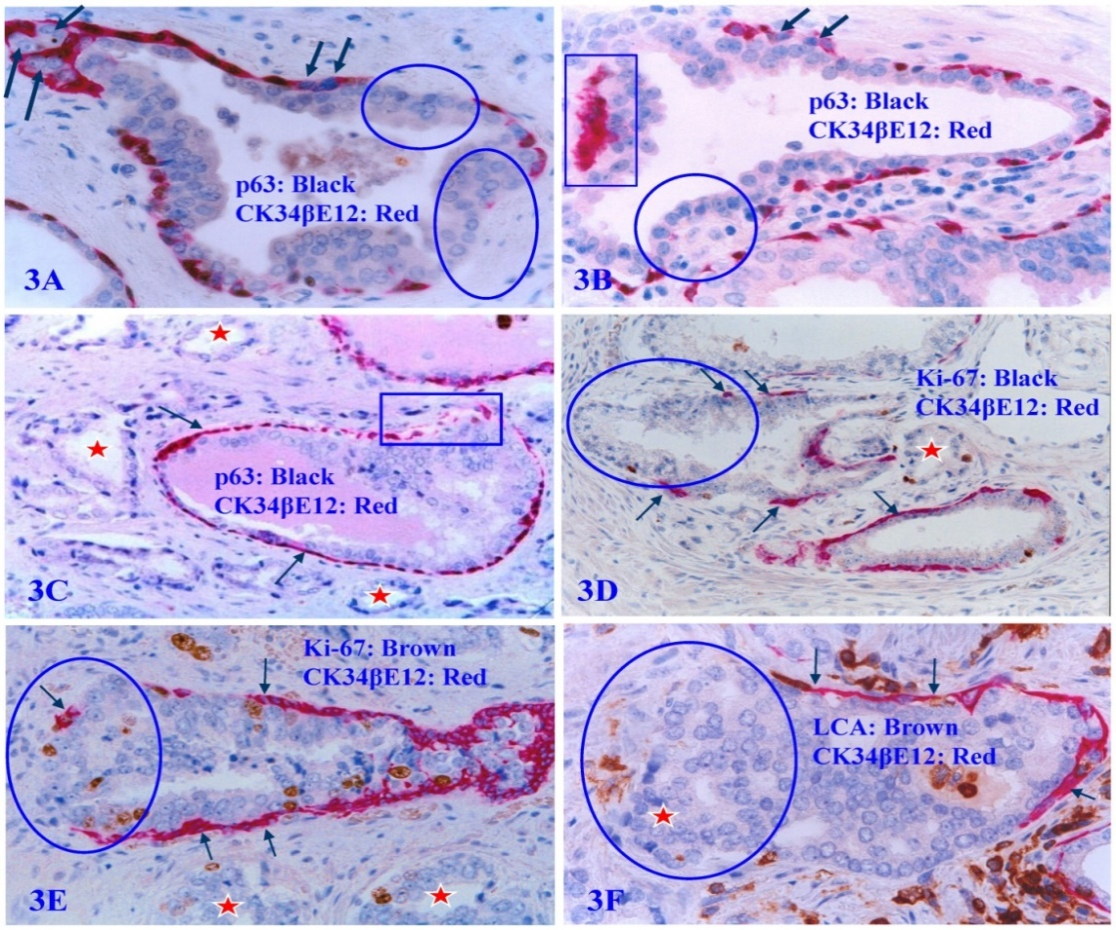
** Focal PBCL senescence or degenerative changes.** Formalin-fixed and paraffin-embedded human prostate gland tissue sections were double immune-stained (see the detailed labeling within individual figures). Circles identify focal PBCL disruptions; Squares identify large clusters of basal cell debris; Thick arrows in A and B identify basal cells with the loss of nuclear p63 expression; Thin arrows identify the residual PBCL; Red stars identify invasive lesions. Please note that the size of the focal disruptions in PBCL varies substantially, and that the epithelial cells overlying focally disrupted PBCL are morphologically more similar to the adjacent invasive cancer cells with a higher proliferation rate than their adjacent counterparts still enclosed by the residual PBCL.

The detailed dynamic cellular kinetics of the human prostate basal cell population remains elusive 55-61. However, distinct senescence or degenerative changes of PBCL are frequently seen in some of normal and malignant prostate tissues. These changes include: focal disruptions (defined as the absence of basal cells resulting in a gap larger than the combined size of at least three basal cells in at least two or more consecutive sections), the loss of nuclear p63 expression, nuclear swelling, cell debris, and large clusters of cell debris. Luminal cells overlying focally disrupted PBCL often show markedly higher cellular density, proliferation rate, and morphology compared to those from their counterparts overlying non-disrupted PBCL (Figure 3).

## The focal PBCL disruption is absolutely needed for tumor invasion

**Figure 4:**
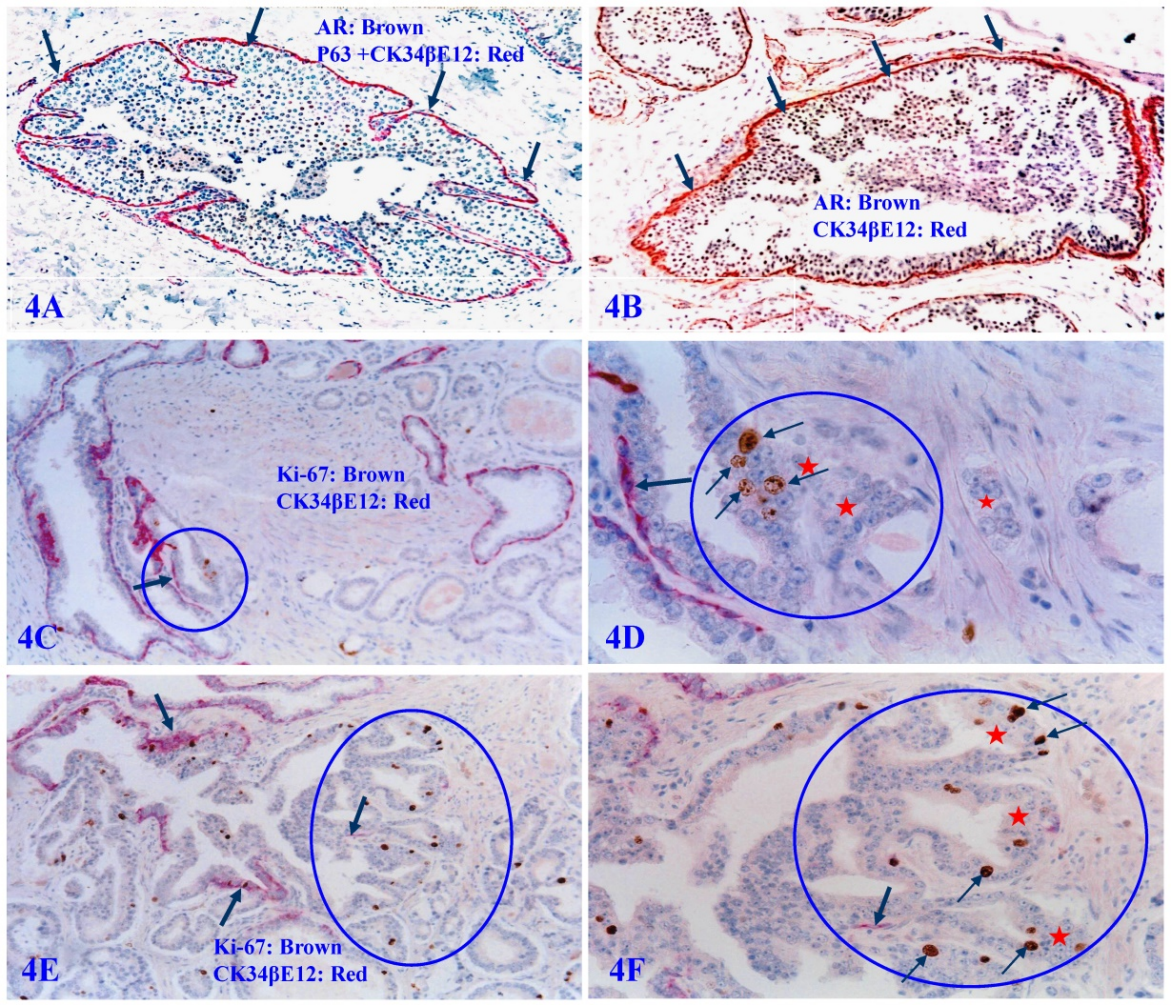
** Focal PBCL disruptions and cancer invasion.** Formalin-fixed and paraffin-embedded human prostate gland tissue sections were double immune-stained with several antibodies (see labeling within each individual picture). Circles identify focal disruptions in PBCL and the overlying epithelial cells. Thick arrows identify the normal or residual PBCL Thin arrows identify proliferating cells. Red starts identify invasive lesions. Please note that the budding cells overlying focally disrupted PBCL have a significantly higher proliferation rate than their counterparts enclosed by the residual PBCL, and that budding cells are immediately adjacent to or in a direct continuity with invasive lesions.

Our previous studies have consistently demonstrated that a focal disruption in a given PBCL is absolutely needed for the initiation of cancer invasion 62-68. If the surrounding FBCL is intact, such tumors can grow to a very large size, but may remain at non-invasive state for many years (Figure 4A and 4B). In a sharp contrast, if the surrounding FBCL is focally disrupted, the cancer invasion could commence at a very early stage, despite a very small size (Figure 4C-4F). Double immunochemical staining with the cell proliferation specific marker Ki-67 and the PBCL specific marker CK34βE12 reveals that all invasion starts at the site of focal PBCL disruptions and a vast majority of proliferating cells are located at or near the site of focal PBCL disruptions. The “budding cells” from focally disrupted PBCL are often immediately adjacent to or in a direct continuity with the invasive cancer cells (Figure 4C-4F).

**Figure 5:**
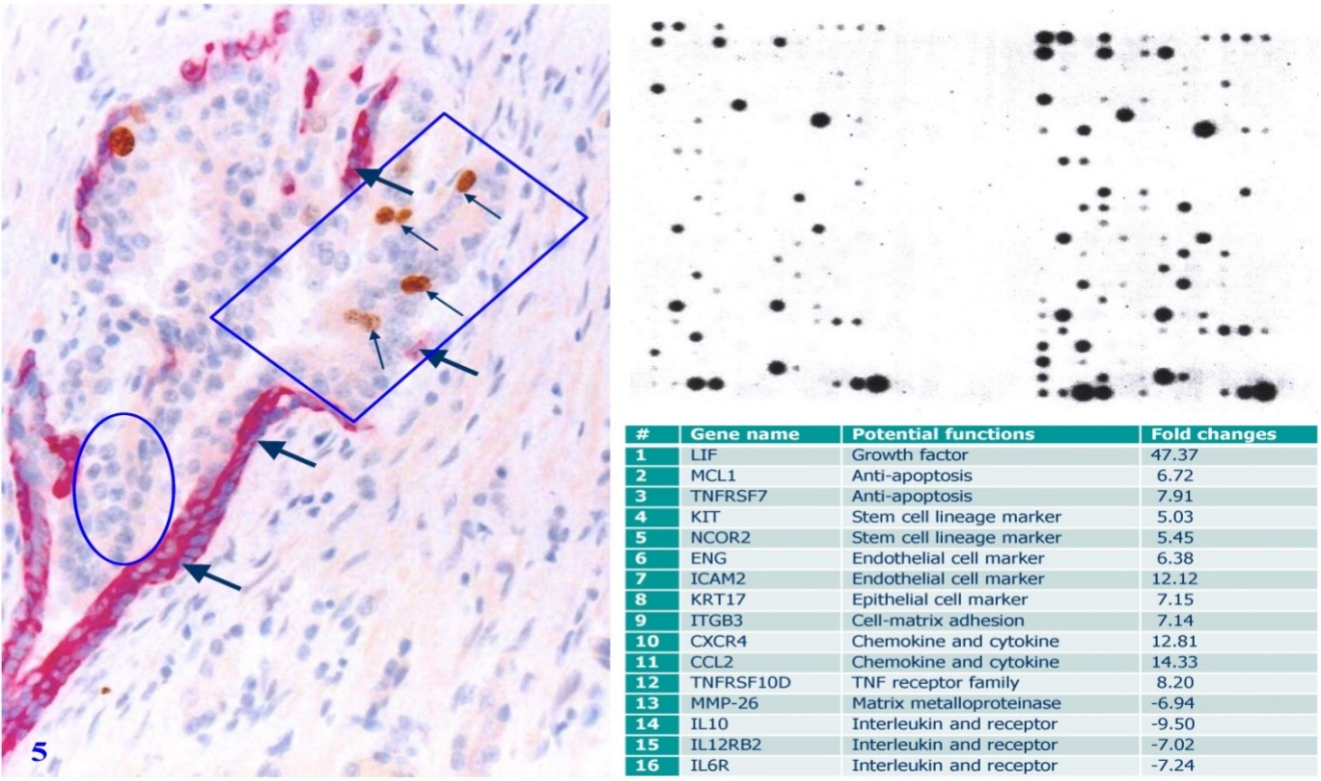
** Gene expression profiling of cells overlying focally disrupted and enclosed by residual PBCL. Formalin-fixed and paraffin-embedded human prostate gland tissue sections were double immune-stained with antibodies for SMA and Ki-67.** Thick arrows identify residual PBCL. Thin arrows identify proliferating epithelial cells. The square identifies epithelial cells overlying focally disrupted FBCL, and the circle identifies epithelial cells enclosed by the residual FBCL micro-dissected for gene expression profiling. Please note that morphologically comparable epithelial cells overlying focally disrupted PBCL and enclosed by the residual PBCL have different profiles of the gene expression.

Selective gene expression profiling of micro-dissected human prostate gland tissue samples has consistently shown that cell clusters overlying focally disrupted PBCL have a significantly higher expression level of cell growth- and invasion-related molecules, including growth factors, stem cell lineage markers, anti-apoptosis-related genes, and endothelial cell makers than their morphologically similar counterparts enclosed by the residual PBCL 66 (Figure 5).

## The PBCL itself is the target of a variety of pathologic alterations

It has been consistently documented that prostate basal cell carcinoma is very rare, accounting for only about 0.01% of the total prostate malignancies 69-73, which strongly suggests that the prostate basal cell population is very unlikely to be a major target of the prostate carcinogenesis. However, it has also been consistently demonstrated that the PBCL belongs to a self-renewal cell population 74-80. For this and other reasons, the PBCL population itself also frequently suffers from a variety of degeneration- and regeneration-related pathologic alterations that includes, but is not limited to the following:

### The loss or significant reduction of nuclear p63 expression in normal appearing PBCL

**Figure 6:**
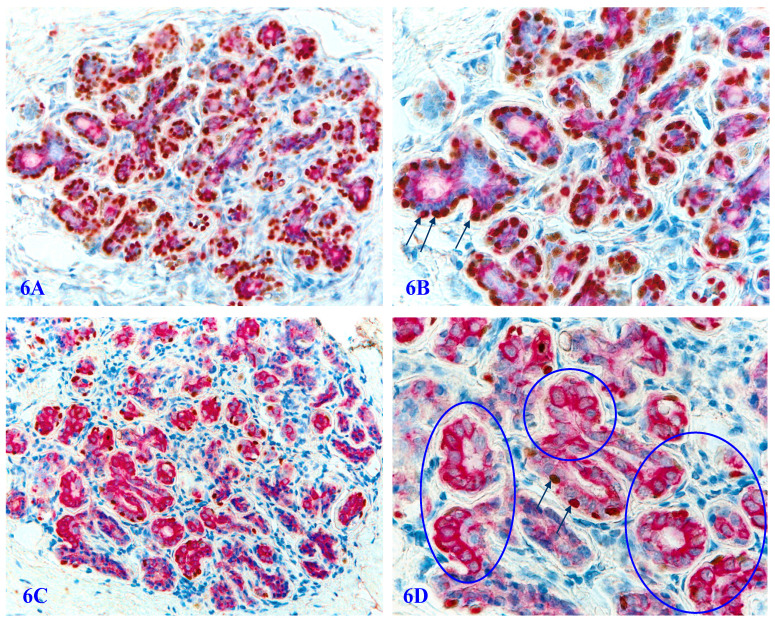
** Significantly reduced basal cell numbers of p63 expression in normal appearing PBCL.** Formalin-fixed and paraffin-embedded human prostate gland tissue sections from two different biopsies with morphologically normal appearing basal cell layers associated with a morphologically normal appearing epithelial cell population were double immune-stained with p63 and Maspin. In the cases one, a vast majority of the normal appearing basal cells display the distinct expression of p63 within the cell nuclei. However, in a sharp contrast in the case two, only around 1-3% of the normal appearing basal cells show the normal localization of p63 expression within the cell nuclei. Circles identify normal appearing basal cells in the case 2, which display a high level of Maspin expression within the cell cytoplasm, whereas they are completely devoid of p63 expression within the cell nuclei despite the fact that the nuclei of these basal cells are clearly visible. 6B and 6D are the higher magnification of 6A and 6C, respectively.

In autopsy, biopsy, and surgically resected human prostate gland tissues, a vast majority of the basal cells in normal or hyperplastic tissues are not only morphologically distinct, but also express a high level of p63 and Maspin, two well-documented tumor suppressors (Figure 6A-6B). However, about 6% to 8% of the cases harbor variable numbers of morphologically normal appearing basal cell clusters that are completely devoid of, or have a significantly reduced cells with p63 nuclear expression (Figure 6C-6D). Prostate cancer patients with such atypical basal cell clusters have a significantly more aggressive clinical courses and worse prognosis 81.

### The loss of the expression of all PBCL specific markers

**Figure 7:**
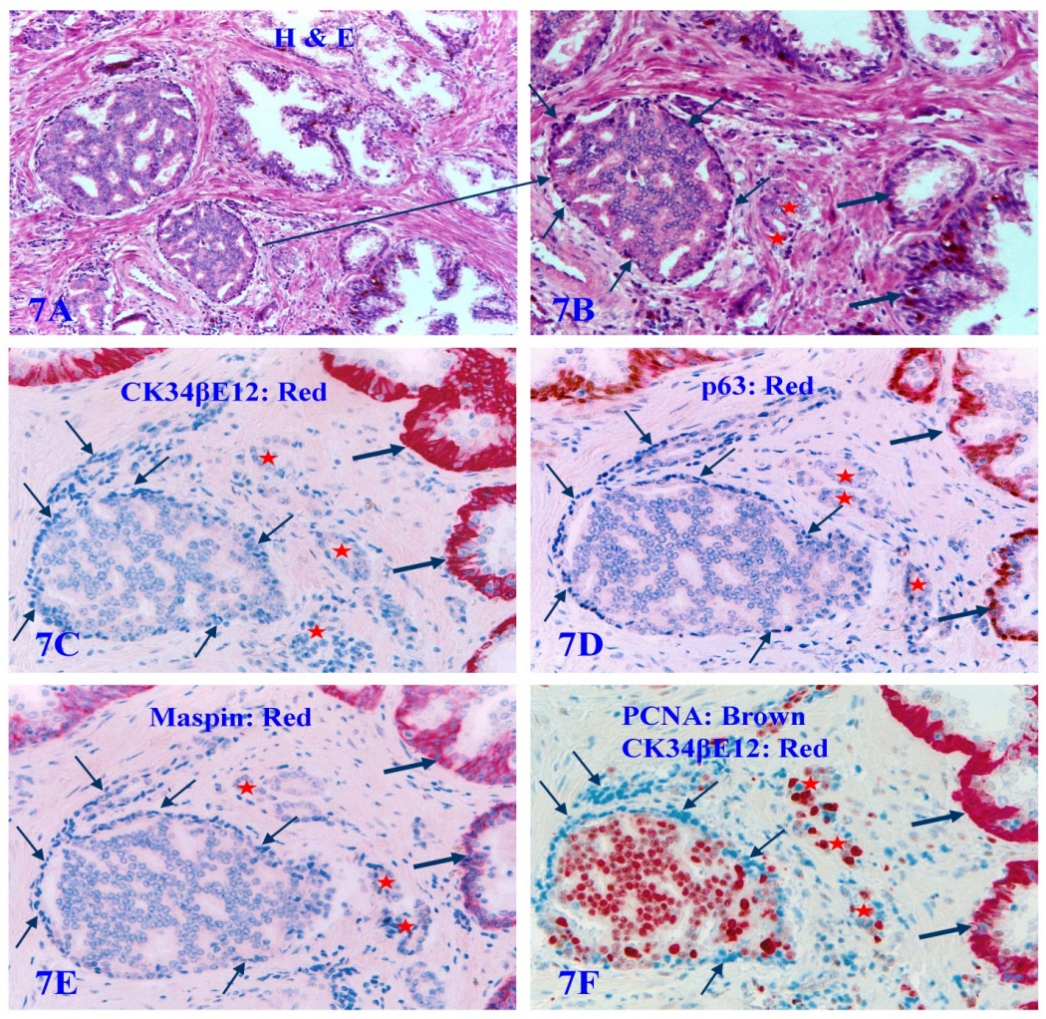
** The lack of all basal cell markers in the PBCL of pre-invasive prostate cancer clusters.** Formalin-fixed and paraffin-embedded consecutive human prostate gland tissue sections from one case were subjected to H&E staining (7A-7B) and IHC staining (7C-7F) for different biomarkers (see detailed labeling within each individual picture). Red starts identify invasive lesions. Thin arrows identify atypical basal cells. Thick arrows identify normal basal cells. Please note that the PBCL overlying a walnut-like tumor nest is morphologically distinct in both H&E and IHC stained sections, but all basal cells are completely devoid of the expression of CK34βE12, p63, and Maspin. In addition, these morphologically distinct basal cells are also completely devoid of the expression of proliferating cell nuclear antigen (PCNA), in a sharp contrast to the enclosed epithelial cells, which are all strongly immunoreactive to PCNA.

About 6% to 8% of the autopsy, biopsy, and surgically resected human prostate tissues also harbor a variable number of atypical pre-invasive cancer cell clusters, in which all the basal cell layers are largely non-disrupted and morphologically distinct, whereas they completely lack the expression of CK34βE12, p63, and Maspin. They are even completely devoid of the expression of the proliferating cell nuclear antigen (PCNA). These pre-invasive cancer cells enclosed by such PBCL are morphologically and immunohistochemically similar to adjacent invasive cancer cells, and have a significantly higher proliferation rate than their adjacent counterparts at the same stage 81 (Figure 7).

### Frequent apoptosis-related alterations

**Figure 8:**
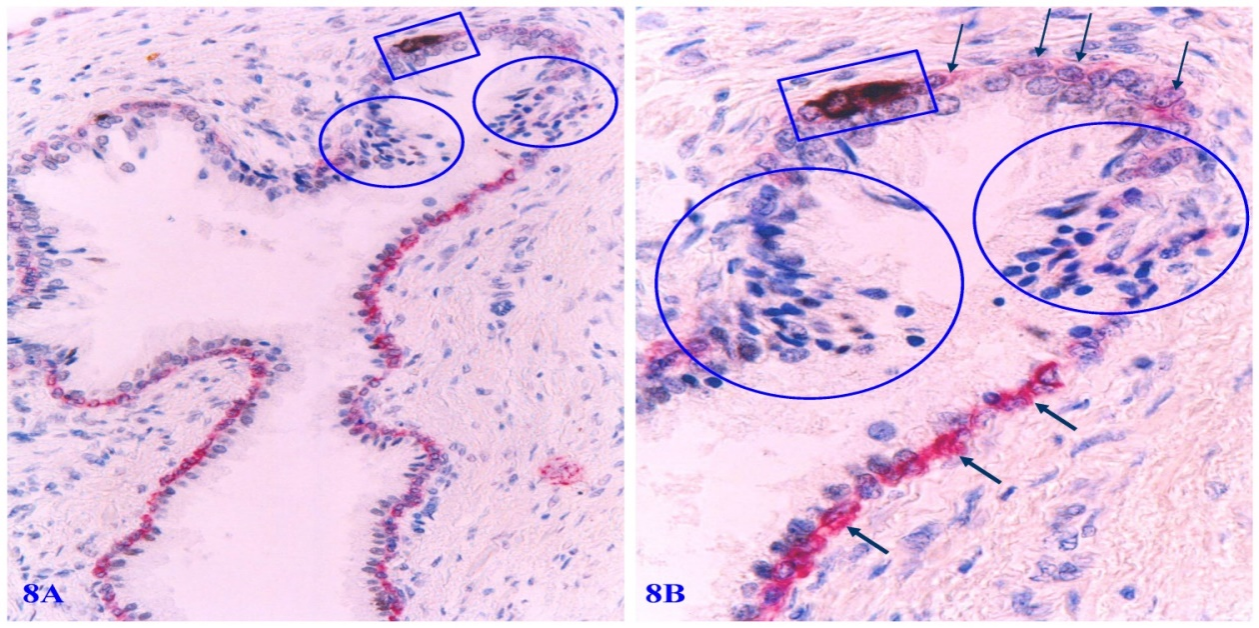
** Apoptosis-related alterations in degenerated basal cells.** Formalin-fixed and paraffin-embedded human prostate gland tissue sections were subjected to the apoptotic assays with a commercially available detection kit, and then, were immune-stained for CK34βE12 to elucidate PBCL. Arrows identify PBCL. Squares identify apoptotic basal cells. Circles identify epithelial cells overlying or near the focally disrupted PBCL.

Distinct apoptosis-related alterations of basal cells are frequently seen in focally disrupted PBCL. Immunohistochemistry-based apoptotic assays clearly demonstrate that a vast majority of focally degenerated basal cells show high levels of apoptosis-related molecules. All apoptotic basal cells are physically located at or near focally disrupted PBCL. Epithelial cells adjacent to apoptotic basal cells have a much higher cell density than their adjacent counterparts (Figure 8).

### The significant infiltration of immune cells

**Figure 9:**
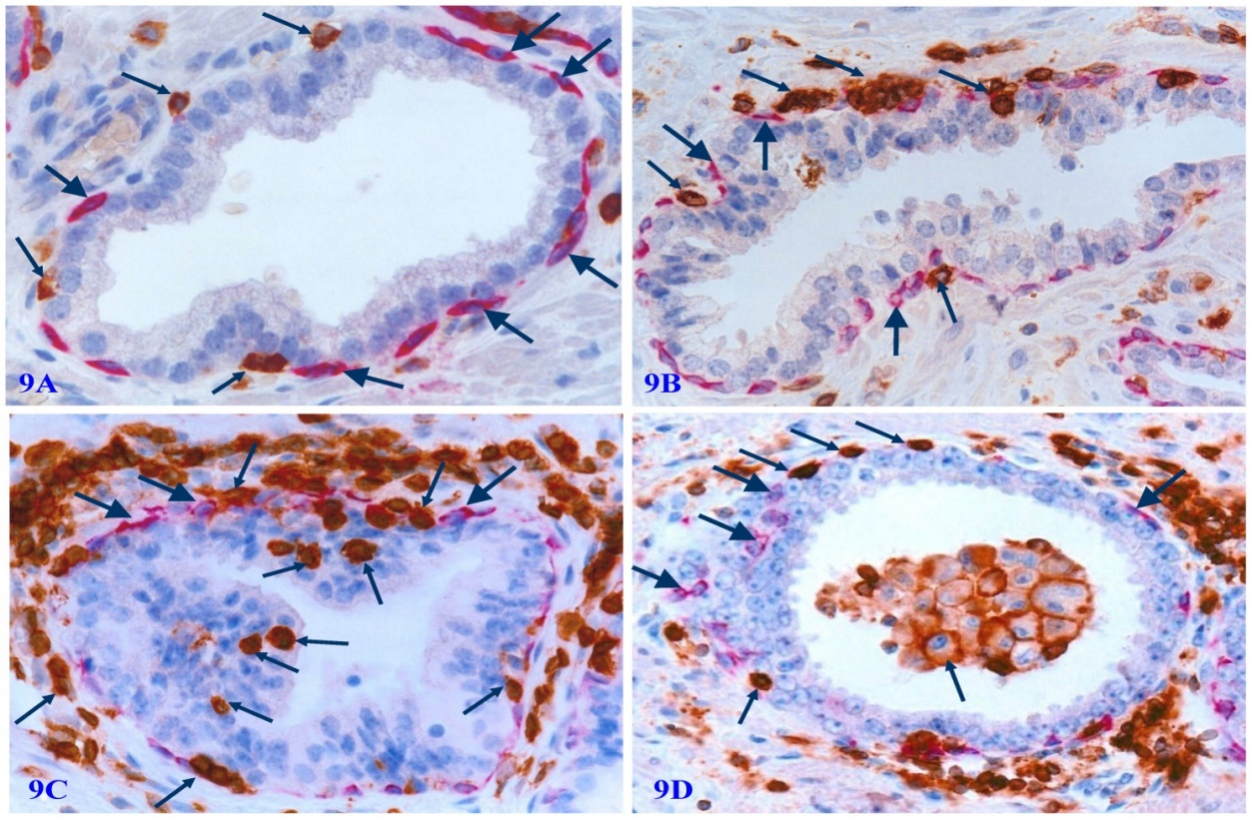
** Physical association of infiltrating immune cells with degenerated basal cells.** Formalin-fixed and paraffin-embedded human prostate tissue sections from four different cases were subjected to double IHC staining with the basal specific marker CK34βE12 and infiltrating immune cell marker (leukocyte common antigen, LCA). Thick arrows identify residual basal cells. Thin arrows identify infiltrating immune cells. Please note that a vast majority of infiltrating immune cells are physically associated with or immediately adjacent to degenerative basal cells. The epithelial component or the acinar and ductal lumen in focally disrupted PBCL often harbor infiltrating immune cells. It is interesting to note that nearly all the normal, benign, and malignant epithelial cells show no distinct sign of degeneration-related alterations.

Degenerated basal cell products appear to act as cytokines to attract infiltration of immune-reactive cells to the physical sites of focally disrupted PBCL, and infiltrated lymphocytes are generally associated with cell debris or morphologically degenerated basal cells (Figure 9).

### The high level of Tenascin C expression in focally disrupted PBCL

**Figure 10:**
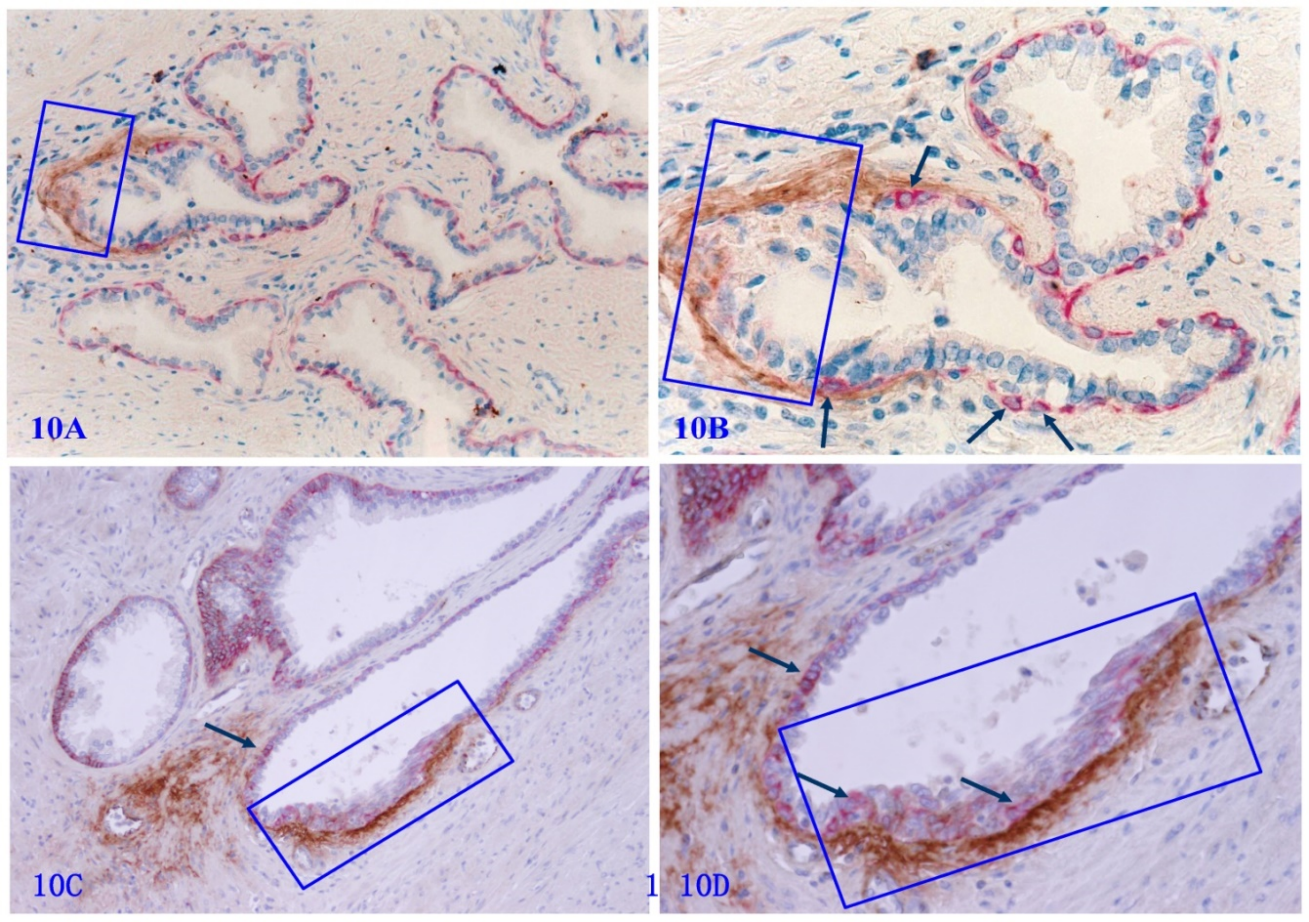
** Tenascin expression at the site of focally disrupted PBCL.** Formalin-fixed and paraffin-embedded human prostate gland tissue sections from two different cases were double immune-stained for CK34βE12 (red) and Tenascin C (brown). Arrows identify residual basal cells. Squares enclose Tenascin C overlying focally disrupted PBCL. Please note that strong Tenascin C positivity is seen only at or adjacent to the site of focally disrupted PBCL. Non-disrupted PBCLs are largely devoid of Tenascin C expression.

Tenascin C is an extracellular matrix glycoprotein, which paves the paths and facilitates the migration and metastasis of prostate cancer cells 82-86. Our previous studies have shown that Tenascin C is highly expressed at the site of distinct degenerative basal cells, and epithelial cells in the vicinity of areas with elevated Tenascin C often lose the cohesion 66 (Figure 10).

Based on above findings, it is apparent that PBCL is not only an essential constitute of the prostate gland, but also an active producer of tumor suppressors, which exert significant decisive influence on adjacent epithelial and stromal cells. In addition, as the PBCL population belongs to a self-renewal population, it has to consistently undergo normal self-replenishment processes to replace aged and injured cells. Consequently, the PBCL population itself also suffers from a wide variety of degeneration- and regeneration-related normal and pathological alterations.

The detailed cellular and molecular mechanisms of focal disruptions or a total loss of the PBCL remains elusive. Furthermore, there is no solid evidence to determine whether the loss of basal cells is a direct trigger for the development of prostate adenocarcinoma, or the loss of basal cells is directly resulted from prostate cancer cells. However, it has been consistently concluded that focal disruptions or a total loss of the PBCL is statistically correlated with the invasion and metastasis of almost all types of prostate cancer (except prostate basal cell carcinoma) 62-73.

Based on above facts, the PBCL appears to be the most effective but largely ignored target for the early detection and intervention of prostate cancer. As the morphologic, pathological, and immuno-histochemical profiles of the basal cell population is far more easily recognizable and definable than its epithelial counterpart, the PBCL appears to be a more easily readable roadmap with the following specific scientific and clinic implications and applications:

- To use Maspin and PSA as independent risk factors for cancer screening. As Maspin is consistently expressed in basal cells 87-90, while the PSA is elevated in virtually all prostate malignancies 91-94, Maspin- and PSA-related signatures in the serum can be utilized for a population-based screening for the early detection of prostate cancer.

- To use p63 as a risk factor for a population-based screening to detect the predisposition of cancer susceptibility or tumor suppressing genes. Since p63 belongs to the p53 tumor suppressor family, and is normally expressed in the nucleus of the basal cells 95,96, an aberrant expression level or subcellular localization of p63 accompanying by an elevated PSA level may signify the predisposition of cancer susceptibility or mutated suppressing genes. Previous studies have clearly demonstrated that loss or cytoplasmic expression of p63 is associated with elevated cancer stem cells, enhanced cell migration and metastasis, and increased mortality in prostate cancer 97-99.
- To use p63, Maspin, and different RNA signatures as biomarkers for the non-invasive detection of early prostate cancer. Previous studies have revealed that p63, Maspin, RNA signatures, and PSA are detectable in the urine samples of prostate cancer patients 100-106. Thus, a statistical comparison of the expression levels of p63, Maspin, and PSA in the urine samples may be used as a non-invasive clinic test for the detection of early prostate cancer.
- To use exfoliated basal cells combined with other urinary markers for early detection of prostate cancer progression and invasion. Previous studies have consistently shown that a variable number of exfoliated cancer cells are detectable in a majority of prostate cancer patients 107-110. As the basal cells are localized at the base of the epithelial cell layer, the detection of exfoliated basal cells in the urine is likely to signify the disruptions of the PBCL and the stromal invasion of the prostate cancer.
- To use the PBCL physical integrity (disrupted vs non-disrupted) combined with PSA test results as a clinic marker for the differentiation diagnosis. As the disruption of the PBCL is a prerequisite for prostate cancer invasion and metastasis, while an elevated PSA level is detectable in both non-invasive and invasive prostate cancer, the physical integrity of the PBCL in patients with an elevated PSA could effectively differentiate between non-invasive and invasive prostate cancer.
- To use the expression of Tenascin in PBCL as a routine clinic test of the prostate biopsy or urine sample. Previous studies have consistently demonstrated that aberrant Tenascin expression is exclusively seen at or near focally disrupted PBCL and is also significantly correlated with prostate cancer invasion and metastasis 66, 82-86. Thus, the assessment of the PBCL-associated Tenascin expression may lead to the identification of the specific cases at increased risk for prostate cancer progression.
- To use Maspin or CK34βE12 as biomarkers to discriminate prostate from non-prostate cancers. Previous studies have shown that (a) high levels of PSA is seen in patients with breast, lung, ovary, liver, kidney, adrenal, skin, salivary, and colorectal cancer 111-113, (b) smoking, asymptomatic inflammation, metformin use, chronic prostatitis can elevate the PSA expression level 114-117, and (c) age, ethnicity, triglyceride level, and BMI can also significantly impact the expression of PSA 118, 119. In contrast, above factors have little impact on PBCL. Thus, normal expression status of Maspin or CK34βE12 in individuals with high levels of PSA may signify non-prostate lesions.
- To use focal PBCL disruptions as a localizer to identify cancer-stem cell clusters/specific precursors of invasive cancer. Our previous studies of multiple cancers have consistently shown that a focal disruptions of tumor capsules selectively facilitate clonal proliferation of overlying cancer stem cells to form distinct cell clusters. These newly formed clusters have significantly higher levels of cancer stem cell markers and invasion and metastasis-related genes than their morphologically comparable counters still enclosed by the non-disrupted tumor capsules 62-66, 120-123 (Figure 4 & 5). It is very likely that these cell clusters may represent the direct precursors of invasive lesions. Thus, micro-dissecting these clusters for further evaluation can potentially lead to the dentification of triggering molecules for the basal cell degenerations, tumor progression, and invasion.
- To use PBCL-associated immune-cell infiltration to monitor the tumor progression and treatment responses. Our previous studies have consistently revealed that the immune-cell infiltration is significantly associated with prostate tumor capsule disruptions which lead to the subsequent invasion and metastasis 62-68. A great number of recent studies have not only confirmed our previous reports and conclusions, but have also consistently shown that immune-cell infiltration is also significantly correlated with the treatment responses in multiple cancer types 62-68, 124-131.
- To use anti-inflammatory drug aspirin or statin to repair the PBCL degeneration-related tumor capsule disruptions. Previous studies have consistently shown that aspirin or statin could significantly alter the immune milieu of prostate and to prevent cancer progression 132-134. Therefore, the administration of aspirin or statin to individuals with focally disrupted PBCL associated with significant infiltration of the immune cells (as shown in Figure 9) and chronic prostatitis could potentially reduce the extent of associated immune cells and facilitate the repairing of focally disrupted tumor capsules.
- To administer stem cell specific molecules, inducers, or stimulators to burst the normal replenishment and the physical integrity of PBCL. Previous studies have suggested that BMP5, Zeb1, CD24, CD44, NANOG, and Nestin are prostate stem cell-specific markers that are essential for the maintenance of the normal replenishment and physical integrity of the PBCL 135-137. Thus, the administration of these molecules or stem cell specific inducers or stimulators to patients at a high risk of prostate cancer progression may offer the promise of more effective approaches for prostate cancer early intervention.
- To use basal cells lacking the phenotypic and proliferation markers as targets to identify novel cell proliferation pathways or cell cycle regulators. As a subset of morphologically distinct and non-disrupted PBCL completely lack the expression of tumor suppressors, phenotypic markers, and cell proliferation specific markers (Figure 7), it is likely that the growth and expansion of these cells are regulated by previously undescribed mechanisms or pathways 138,139. Therefore, microdissection of these PBCL for gene expression profiling may lead to the identification of novel cell proliferation pathways and novel cell cycle regulators.

In summary, above findings and analyses strongly suggest that the PBCL is the most effective but largely ignored target for the early detection and intervention of human prostate cancer. It is apparent that the human tissue-derived basic research data may provide a more realistic roadmap that allows to observe the direct interactions among different cell types and to avoid the potential misleading from *in vitro* and animal studies.
